# High Prevalence of Anemia in Children and Adult Women in an Urban Population in Southern Brazil

**DOI:** 10.1371/journal.pone.0068805

**Published:** 2013-07-29

**Authors:** Lucia Mariano da Rocha Silla, Alice Zelmanowicz, Ingrid Mito, Mariana Michalowski, Tania Hellwing, Marco Antonio Shilling, João Ricardo Friedrisch, Christina M. Bittar, Cristina Arthmar Mentz Albrecht, Elaine Scapinello, Claudia Conti, Marcia Arthmar Mentz Albrecht, Letícia Baggio, Annelise Pezzi, Bruna Amorin, Vanessa Valim, Laura Fogliatto, Alessandra Paz, Claudia Astigarraga, Rosane Isabel Bittencourt, Gustavo Fischer, Liane Daudt

**Affiliations:** 1 Hematology and Bone Marrow Transplantation, Hospital de Clínicas de Porto Alegre, Porto Alegre, Rio Grande do Sul, Brazil; 2 Federal University of Rio Grande do Sul, Porto Alegre, Rio Grande do Sul, Brazil; 3 Associação dos Amigos da Hematologia do Hospital de Clinicas de porto Alegre, Porto Alegre, Rio Grande do Sul, Brazil; 4 Laboratory of Cell Culture and Molecular Analysis of Hematopoietic Cells, Hospital de Clínicas de Porto Alegre, Porto Alegre, Rio Grande do Sul, Brazil; 5 Fundação Faculdade Federal de Ciências Médicas de Porto Alegre, Porto Alegre, Rio Grande do Sul, Brazil; 6 Federal University of Pelotas, Pelotas, Rio Grande do Sul, Brazil; 7 University of Passo Fundo, Passo Fundo, Rio Grande do Sul, Brazil; Aga Khan University, Pakistan

## Abstract

This population-based study was designed to detect the prevalence of anemia in a healthy population of children (18 months to 7 years) and women (14 to 30 years) tested in 2006–2007 in the state of Rio Grande do Sul, Brazil as part of an effort to tackle this massive problem that still affects so many people in the XXI century. Anemia was defined according to the WHO. Capillary blood was measured and socioeconomic status was determined according to the *Brazilian Association of Market Research Agencie*s. The median prevalence of anemia in 2198 children was 45.4% and in 1999 women 36.4%. Anemia decreased with age during childhood; although significantly more prevalent in lower classes individuals, it was also high in the upper classes. There are indirect evidences that the lack of iron supplementation and/or iron fortified food may play a role in it. Professionals and society wise measures of education have to be implemented in order to address possible biologic factors involved in childhood psychosocial development in southern Brazil.

## Introduction

There is increasing evidence that micronutrient deficiencies play a central role in preventing children from achieving their full developmental potential and compromising irreversibly their cognitive development [1]–[3]. Anemia is a global concern [4] and represents an ongoing challenge worldwide [5] since human development and social equality are also depending on an adequate micronutrient intake early in life [6]. A recent systematic review on all studies on the prevalence of anemia in Brazil has shown that for children under the age of 5 the median anemia prevalence in the entire country is 53% [7]. Surprisingly, there is also evidence for an increasing prevalence of anemia and obesity, which has been attributed to a “nutrition transition” observed in Brazil [8]. There are few published studies on the prevalence of anemia in fertile, non-pregnant women in Brazil. According to a National Surveillance published in 2006 by the Brazilian Health Ministry, the prevalence of anemia in this population is 25 to 30% [9].

Of the children referred to the Pediatric Hematology out-patient clinic in the Hospital de Clinicas de Porto Alegre (HCPA), the University Hospital of the Federal University of RS (UFRGS), an estimated 40% had iron deficiency anemia (IDA). Based on this and on the findings mentioned above, we decided to launch an educational program called “Children without Anemia in RS”, to draw attention to the prevalence of anemia and to establish measures to prevent and treat it. As part of this program, we ran a population based study on anemia prevalence and its relation to socioeconomic status (SeS) in the state of Rio Grande do Sul (RS) which we report here.

Rio Grande do Sul is Brazil’s most southern state with nearly 11 million inhabitants, a GDP of R$193 billion (∼ USD $ 107 billion) and a life expectancy of 75.3 years [10]. Being a subtropical area, the commoner tropical diseases are rare, the prevalence of hemoglobinopathies is relatively low [11]–[13] and the childhood mortality rate is 8.19 per 1000 live births, mostly related to low birth weight [14].

## Materials and Methods

### Prevalence Study Population

We conducted a cross-sectional study on the prevalence of anemia in children living in urban areas, aged 18 months to 6 years, and women aged 14 to 30 years in the state of RS, southern Brazil, in 2006–2007. The inclusion criteria for children required them to be apparently healthy without any known disease. The women were required not to be pregnant, not puerperal, not related to the children tested and not to have had a spontaneous abortion in the prior 6 months. It was recommended that that the research team should not test siblings or mother and child. However, there is no guarantee that this criterion has been always met. The complexity of such a study with data collected all over the state by many students rendered a long lasting analysis in order to get the most out of it.

### Sample Size Definition And Population Assessment

The number of individuals tested in each state region was based on the population density of the particular region and coincides with the 18 Regional Councils for Health of the State Health Department. For an estimated anemia prevalence of 30%, the projected sample size for this survey was 645 individuals for both, children and women, considering 18 clusters. The estimates assumed an average error of 10% and a loss of 20%. In an attempt to minimize sampling biases, and to diffuse the issue of anemia in the state, the sample size was tripled. In order to test a “healthy” population we relied on a non-governmental organization – Pastoral da Criança [15], which is known worldwide for its work with the poor and for their assistance technique which is based on an individual (Leader) from the community who visits, at least once a month, every household in that community. The data were collected at one particular community depending on their Leader’s availability and the possibility of grouping the target population at one site, at that particular point in time. Informed consent, collection of demographic data, socioeconomic status, capillary blood tests, and completion of relevant documentation were carried out by 100 medical students from all 10 Medical Schools scattered over the state. To be eligible, the student had to give a series of seminars about anemia at their own Medical School and attend the several meetings planned to take place in their region.

### Demographic Data

For children we recorded gender, age, age of the mother, number of siblings and skin color. For women, age, number of previous offspring and skin color were recorded. Skin color was recorded as white, black and other (mixed – ethnicity), according to self-reported perception for women, and for the children according to the views of the mother or of other persons responsible for the children.

### Socioeconomic Status Definition

Socio-economic classification was done by filling out a questionnaire from a population based classification test designed by the Brazilian Association of Market Research Agencies and utilized by the government to classify the Brazilian population into 5 levels: A to E – A being the highest and E the lowest socioeconomic status [16].

### Blood Testing

Hemoglobin concentration was determined by testing capillary blood using the HemoCue System (Leo Diagnostics, Helsingborg, Sweden) [17] and anemia was defined in accordance with the World Health Organization (WHO): <11 g/dL and <12 g/dL for children and women, respectively [5].

### Statistical Analysis

The statistical applications WINPEPI and Statistical Package for the Social Science (SPSS) version 18 were used for the analysis and calculation of the sampling. In the analysis, we calculated the crude prevalence ratio and the adjusted prevalence by means of Poisson regression with a 95% confidence interval. A city was considered as a variable cluster.

### Ethical Aspects

This study was approved by the Ethics Committee of HCPA under number 01–364. Written informed consent was obtained from every participant, caretakers or guardians for the children. The signed informed consent was stored in a protected location and the data analyzed anonymously according to Declaration of Helsinki for human studies.

## Results

From the total of 2376 children and 2072 women tested, it was possible to evaluate 2198 and 1999, respectively with a loss of 7.5% for children and 3.5% for women due to incomplete forms. Of the evaluable population of children, the median age was 51 months, 1132 (51.5%) were girls, the median age of their mothers was 29 years, and the median number of siblings per child was 2 (zero to 16). Of the evaluable fertile non-pregnant women tested, the median age was 22 years (14 to 30 years of age), the median number of previous offspring was 1.4, ranging from zero to 12, and 715 (36%) were nulliparous. Among the 363 adolescents (with 16 years of age or less), there were 31 with one, 4 with two and 1 with three previous pregnancies; of the older women, 76% (1248/1636) have had at least one pregnancy.

### Prevalence of Anemia

The prevalence of anemia for children was 45.4%(IC95% 43.3%–47.5%) and 36.4%(IC95% 34%–38.3%) for women. The median hemoglobin concentration for children and women was 9.9 g/dL (4.0 to 10.9 g/dL) and 12.5 g/dL (5.2 to 17.8 g/dL), respectively. According to the age group the prevalence of anemia for children under 23 months and older than 72 months was 76% and 31%(Table 1). For women there was no correlation between age and anemia prevalence (Table 2). Of the nulliparous women anemia affected 38.2% (125/327) and 34.8% (135/388) of those with 16 years of age or less and older, respectively (p = 0.342). For women that have had at least one pregnancy 47.2% (17/36) of the adolescents and 36.2% (425/1248) of the older were anemic (p = 0.176).

**Table 1:** Univariate analysis of predicting factors for anemia in children in Rio Grande do Sul, Southern Brazil, 2006–2007.

Characteristics	N(2198) anemic/total	Prevalence (%)	Gross PR (CI 95%)	P-value
Gender*				
Female	486/1109	43.8%	1	
Male	510/1077	47.3%	1.081(0.986–1.184)	0.098
Age in months				
18 to 23	97/127	76%	2.500(2.042–3.063)	0.000
24 to 35	260/422	62%	2.017(1.662–2.448)	0.000
36 to 47	196/446	44%	1.439(1.170–1.769)	0.001
48 to 59	176/438	40%	1.316(1.065–1.626)	0.011
60 to 71	187/490	38%	1.249(1.012–1.543)	0.038
≥72	84/275	31%	1	
SeS				
A and B	69/201	34.3%	1	
C	347/836	41.5%	1.209(0.983–1.488)	0.073
D	447/933	47.9%	1.396(1.140–1.709)	0.001
E	132/219	60.3%	1.756(1.410–2.186)	0.000
	9 missing			

SeS = socioeconomic status,

* 12 missing.

**Table 2:** Univariate analysis of the predicting factors for anemia in women and crude PR of anemia according to age, social class and skin color in RS, Southern Brazil, 2006–2007.

Characteristics	N (1999)	Prevalence(%)	Crude PR(CI 95%)	P-value
SeS*				
A and B	100/318	31.4%	1	
C	292/847	34.5%	1.096(0.909–1.322)	0.335
D	267/680	39.3%	1.249(1.035–1.506)	0.020
E	68/152	44.7%	1.423(1.119–1.808)	0.004
Skin color #				
White	416/1220	34.1%	1	
Black	182/414	44%	1.289(1.128–1.474)	0.000
Other	119/336	35.4%	1.039(0.881–1.224)	0.651
Age				
14 to 17 years	183/482	37.9%	1.231(0.891–1.701)	0.209
18 to 19 years	88/232	37.9%	1.229(0.871–1.735)	0.240
20 to 24 years	212/620	34.2%	1.108(0.803–1.529)	0.531
25 to 29 years	217/571	38%	1.232(0.894–1.697)	0.202
≥30 years	29/94	30.8%	1	

SeS – socioeconomic status,

* 2 missing,

# 29 missing.

### Anemia Crude Prevalence Ratio (PR) And Predicting Factors For Anemia In Children

Table 1 shows the univariate analysis of predicting factors and crude PR for anemia in children for the independent variables: sex, age, social class and skin color. There was a statistically significant association for the age category ranging from 18 months to 59 months (p<0. 05). The crude PR of anemia in children between 18 and 23 months was 2.5 (IC 95%; 2.042–3.063), which represents double the value found for children aged 72 months. Children belonging to D and E SeS were 39.6% and 75.6% more anemic than the ones belonging to AB SeS, respectively. Black skin color was predictive of anemia (p<0.001). The crude PR of anemia was found to be 31.2% higher in black children than in white. Of the 425 black children tested, 235 were found to be anemic, which shows a prevalence of 55.3% in this population.

### Anemia Crude Prevalence Ratio (PR) And Predicting Factor For Anemia In Non-Pregnant Women

Table 2 shows the univariate analysis of predicting factors for anemia and its crude PR in women according to age, social class, and skin color. For the variable age, no significant association was found in any category. Women belonging to SeS class D and E were 24.9% and 42.3% more anemic than the ones in Class AB, respectively. A significant association (p<0,001) was also observed for black women, with a gross PR of 28.9% when compared to white women. Unfortunately, the age at first pregnancy was not recorded precluding further conclusions.

In the Hierarchic Multiple Poisson Regression (Table 3) the association of anemia with age in children, remains significant for the 18 to 59 months category with younger age as an independent risk factor for anemia. Belonging to the D and E SeS and being black were independent risk factors for anemia in children as well. The multivariate analysis for independent risk factors for anemia in women (Table 4) also showed black skin color and lowers SeS (class D and E) as independent risk factors.

**Table 3:** Hierarchic Multiple Poisson Regression analysis of the predicting factors for anemia in children, RS, Southern Brazil, 2006–2007.

Characteristics	Adjusted PR (95% CI)	p-value*
Age		
18 to 23 months	2.448(1.812–3.307)	0.000
24 to 35 months	1.917(1.450–2.533)	0.000
36 to 47 months	1.400(1.123–1.745)	0.003
48 to 59 months	1.246(1.013–1.533)	0.037
60 to 71 months	1.147(0.968–1.360)	0.113
72 months total	1	
Social Class		
A and B	1	
C	1.190(0.889–1.593)	0.241
D	1.236(0.973–1.569)	0.082
E	1.511(1.159–1.971)	0.002
Skin Color		
White	1	
Black	1.210(1.050–1.396)	0.009
Other	0.977(0.856–1.114)	0.856

PR: Prevalence ratio; SeS: socioeconomic status.

* Hierarchic multiple poisson regression with level of significance<0.05; interval with 95% confidence.

**Table 4:** Hierarchic Multiple Poisson Regression analysis of the predicting factors for anemia in women, RS, Southern Brazil, 2006–2007.

Characteristics	N(1999) Anemic/total	Prevalence (%)	Adjusted PR (95% CI)	P-value
Skin color*				
White	416/1220	34.1%	1	
Black	182/414	44%	1.254(1.095–1.436)	0.001
Other	119/336	35.4%	1.011(0.856–1.194)	0.896
SeS#				
A and B	100/318	31.4%	1	
C	292/847	34.5%	1.078(0.893–1.302)	0.434
D	267/680	39.3%	1.212(1.001–1.466)	0.048
E	68/152	44.7%	1.328(1.040–1.697)	0.023

PR: Prevalence ratio; SeS: socioeconomic status.

Hierarchic multiple Poisson regression with level of significance <0.05; interval with 95% confidence,

* 29 missing,

# 2 missing.

## Discussion

In this study, 45.4% (CI 95%; 43.3%–47.5%) of the 2198 children between 18 months and 7 years old and 36.4% (CI 95%; 34%–38.3%) of the 1999 non-pregnant women aged between 14 and 30 years old in the state of RS were found to be anemic in 2006–2007. Anemia affected 76% of the children under 24 months old, and this prevalence gradually declined to 31% for children over 72 months old. Although there were cases in which the anemia was severe, in most of our anemic population the anemia was mild; the median hemoglobin concentration was 12.4 g/dl and 9.9 g/dL for women and children, respectively. Lower SeS classes were an independent risk factor for anemia for both children and women, but anemia was also identified in the upper classes impressive (Table 1 and 2); in the upper SeS it affected 34.3% of children and 31.4% of adult women.

The overall prevalence of 36.4% of anemia in fertile women in our study was higher than in the country [9], and the 21.9% to the 19.2% reported by others in the same region [18]. It should however be pointed out that the comparison of our data with theirs is hampered by the inclusion in their study of women with ages from 20 to 60 years old. As can be seen at Table 2, anemia equally affected women of all ages. We could speculate that untreated adolescents evolve to be adults with anemia aggravated by pregnancies, since 76% of the women older then 16 years had already had at least one pregnancy.

A national program of iron supplementation for children from 6 to 18 months of age and for pregnant women was launched in 2005 [19] and its implementation have had several caveats with low adhesion among other reasons because of gastrointestinal intolerance and lack of enforcement [20]. As for iron fortification of the flour, established by law in Brazil in 2004, its effect on anemia in preschool children, in RS, was found to be irrelevant due to inadequate flour intake and/or low bioavailability of dietary iron [21]. In spite of that, those are the first national effort to overcome the deleterious effects of micronutrient deficiency in pregnancy and childhood in Brazil. Because of such a recent effort, awareness to that is still been built all over the country.

The prevalence of anemia we found in children accords with that shown for the whole of Brazil [7]. In RS, in a result similar to ours, in 2004 in the city of São Leopoldo, metropolitan region of Porto Alegre, RS, a prevalence of 63.7% of anemia was found in children from 12 to 16 months of age [22]. The inverse association of anemia and age we found has been described in several regions of the country [23]–[27] as well as in RS [22], [28]. Although we did not attempt to investigate the cause of anemia in our sample, in the aforementioned study [22] including 397 children, 95% of the 63.7% of anemic individual were iron deficient, and this deficiency as well as the prevalence of anemia declined with age. Since their study population belongs to the same region as ours, we can probably assume that iron deficiency might play a role in our study population as well. Stunting was recently evaluated in RS in a population of children covered by the Food and Nutritional Surveillance System (SISVAN) [29] and was found in 7.1% of a population of 63.320 children fewer than 10 years of age. Interestingly, 8.4% of the children were overweighed. The authors concluded for the coexistence of high overweight and stunting rates in this age group. Neri [30], referring to the steady decline of stunting in Brazil, and trends to a rapid increase in overweight and obesity for adolescents and adults, attributed these findings to a better income distribution due, among other reasons, to a major expansion of cash transfer programs for poor families [31].

Hemoglobinopathies could have explained the anemia we observed but this seems to be an unlikely explanation. RS did indeed receive immigrants from Northern Italy (Piedmont) [32] but in practice severe thalassaemia cases are rare in RS. In 117.320 newborns in RS screened for hemoglobinopathies in the first few days of life a hemoglobin variant was found in 1.4% [12]. The most frequent abnormality was sickle cell trait (1.342 newborns). As for sickle cell anemia (SCA), the most prevalent hemoglobin abnormality in Brazil [33], there are only about one thousand patients in RS, one out of 11.000 newborns [12].

As has been described by others in the same region of the country [34]–[35], colored individuals of all ages and SeS were at greater risk for anemia. In the United States, after removing alpha-thalassemia, iron deficiency, renal insufficiency or sickle cell trait, African Americans still had lower Hb compared to the white population [36]. There is no similar study in Brazil.

Although in our study we only included children older then 18 months of age, we could speculate that the very high prevalence of anemia detected in children between 18 and 24 moths of age (76%) is a reflection of early life nutrition.

In RS, a nested cross-sectional study was conducted in a contemporary cohort in the city of Porto Alegre [37]. The authors found that the incidence rate of bottle-feeding was 21.3% for newborns at Day 7, and 46.9% for infants at Day 30 of life, suggesting the early introduction of cow milk. The use of cow’s milk in lieu of other foods rich in bio available iron was shown to be a risk factor for childhood anemia in several studies in Brazil and abroad [38]–[42].

Low SeS, known to be a risk for anemia, was also an independent risk factor for anemia in women and children in our population, as was also demonstrated by others in the same region [43]. Although to belong to the upper social stratus was not an independent risk factor, anemia was also very prevalent in the upper social classes for both women and children (Tables 1 and 2).

In conclusion, anemia is a public health issue in RS, affecting all social stratus, fertile women at all ages and 76% of the children from 18 to 24 months of age. National efforts to overcome micronutrient deficiencies are recent and still in progress in Brazil. The evidences brought by our study underline the need of implementing educational programs such as “Children without Anemia in RS” designed to built awareness to the problem among physicians, nurses, nutritionists and professionals involved in primary health care, as well as to the society, in general.
